# Nitrofurantoin-Induced Pulmonary Toxicity: Always Review the Medication List

**DOI:** 10.7759/cureus.9807

**Published:** 2020-08-17

**Authors:** Caitlin Batzlaff, Matt Koroscil

**Affiliations:** 1 Internal Medicine, Brooke Army Medical Center, Fort Sam Houston, USA; 2 Department of Pulmonary and Critical Care Medicine, San Antonio Uniformed Services Health Education Consortium, Joint Base San Antonio-Fort Sam Houston, USA

**Keywords:** nitrofurantoin, toxicity, pulmonary, diffuse lung disease, drug-induced lung disease, chronic dyspnea on exertion

## Abstract

Use of nitrofurantoin for uncomplicated cystitis and recurrent urinary tract infections is common practice. While the majority of patients tolerate this medication without issue, it is important to be cognizant of adverse reactions, as these can impact patient’s quality of life. Nitrofurantoin-induced pulmonary toxicity is a rare side effect that can present with various clinical manifestations, imaging abnormalities, and pathologic findings. We describe a case of chronic pneumonitis in a patient on suppressive nitrofurantoin therapy presenting with dyspnea and hypoxemia.

## Introduction

Nitrofurantoin is an oral antibiotic that functions to disrupt normal bacterial cellular function. It does this by altering ribosomes and subsequently changing protein synthesis, metabolism, and synthesis of deoxyribonucleic acid (DNA), ribonucleic acid (RNA), and the cell wall. Nitrofurantoin is also bactericidal in urine providing additional clearance of bacteria [1]. This leads to a limited resistance profile and overall mild “collateral damage” profile according to the Infectious Diseases Society of America [2]. For these reasons, it is recommended as first-line, empiric coverage for a patient presenting with acute uncomplicated cystitis. Its use is also commonly extrapolated for the prevention of recurrent urinary tract infections [3,4]. Recurrent urinary tract infections are defined as two or more infections in a six month period or three or more infections in a twelve-month period [3]. However, with prolonged use of nitrofurantoin, there have been reports of both pulmonary and hepatotoxicities [5].

While nitrofurantoin-induced pulmonary toxicity is well known in the literature, it is not commonly included in the initial differential for chronic exertional dyspnea with hypoxemia. Here we describe a case of worsening chronic dyspnea in a patient on preventative nitrofurantoin therapy for recurrent urinary tract infections.

## Case presentation

A 70-year-old female presented to the pulmonary clinic for repeat evaluation of chronic dyspnea on exertion. Her pertinent past medical history included obesity, obstructive sleep apnea, and non-obstructive coronary artery disease. Social history is notable for remote tobacco use with cessation at age 18. She had been seen for similar complaints approximately five years ago with a normal work up to include spirometry, six-minute oxygen desaturation test, chest X-ray, echocardiogram, left and right heart catheterization, and high-resolution CT chest (HRCT). At that time, she had a borderline positive methacholine-challenge test (noted to have a 28% reduction in forced expiratory volume in one second (FEV1) with the 16 mg dose of methacholine). She was trialed on inhaled corticosteroid therapy without improvement, as well as a short-acting β-agonist with subjective relief.

**Figure 1:**
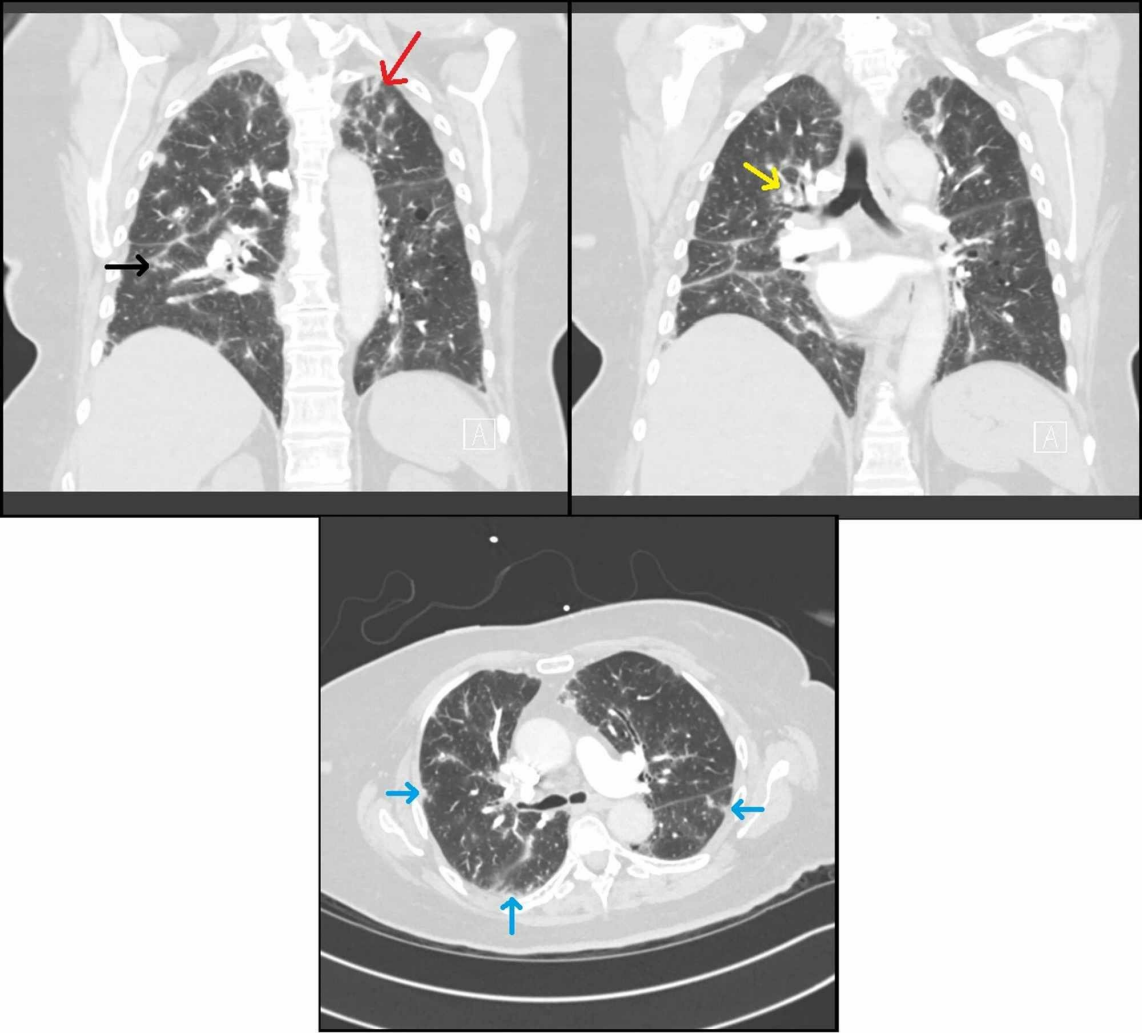
CT imaging on presentation A) Coronal CT of the chest noting subpleural ground glass opacities (black arrow) and slight upper lobe predominance of ground glass opacities (red arrow). B) Coronal CT of chest noting peribronchovascular ground glass opacities (yellow arrow). C) Axial CT of chest demonstrating diffuse nodular ground glass opacities in a subpleural (blue arrows) distribution.

In February 2018, she presented to the emergency department with pre-syncopal symptoms, acute on chronic worsening of her dyspnea, and falls. The patient was afebrile and normotensive, but hypoxemic requiring supplemental oxygen via nasal cannula. Physical examination revealed bilateral fine crackles. CT pulmonary angiogram during admission revealed new diffuse peribronchovascular ground-glass opacities with sub pleural involvement, multiple perifissural nodules, and right hilar lymphadenopathy (Figure 1). 

The pulmonology service was consulted during her admission and she underwent bronchoscopy with bronchoalveolar lavage (BAL) as well as transbronchial biopsies. BAL showed 43% lymphocytes, 16% polymorphonuclear neutrophils, 2% eosinophils. The cluster of differentiation (CD)4/CD8 ratio was ~1 (normal). She had negative bacterial and acid fast bacilli cultures on BAL. The patient had a respiratory polymerase chain reaction (PCR) panel which was negative for influenza A/B and other common respiratory pathogens including Mycoplasma pneumoniae and Chlamydia pneumoniae. Autoimmune serologic testing was negative. Her transbronchial biopsy showed normal alveolar tissue, thought to be a sampling error. The patient was not initiated on corticosteroid therapy. Prior to discharge, the patient failed an oxygen desaturation test (Table 1). She was discharged home in stable condition, with supplemental oxygen therapy, and outpatient pulmonary follow up.

**Table 1:** Oxygen desaturation studies

Time Frame	SpO2 at rest on room air	Ambulatory SpO2	Required O2 flow to maintain SpO2
4 years prior to presentation	96%	Nadir of 92%	No supplemental oxygen required
On discharge from hospital	90%	Dropped to 88% after 3 minutes walked (total ambulation of 100 feet)	2 liters/minute to achieve SpO2 92% in 2 minutes

On follow up, she provided interval history of recurrent urinary tract infections and chronic nitrofurantoin use. She had been on nitrofurantoin for approximately 15 months. She was given a working diagnosis of organizing pneumonia, a subset of interstitial lung disease. Nitrofurantoin was discontinued. Subsequent pulmonary function tests (PFTs) showed a greater than 10% improvement in DLCO that paralleled both symptomatic and radiographic improvement (Table 2).

**Table 2:** Pulmonary function test results FVC: forced vital capacity; FEV1: forced expiratory volume in one second; TLC: total lung capacity; DLCO: carbon monoxide diffusing capacity.

Time Frame	FVC/%	FEV1/%	Ratio	TLC	DLCO/% predicted
3 months after presentation	2.76/98	2.23/105	81	4.36	8.6/38
9 months after presentation	2.93/107	2.23/108	76	3.60	10.0/50
14 months after presentation	3.01/111	2.40/117	80	4.61	11.1/51

**Figure 2:**
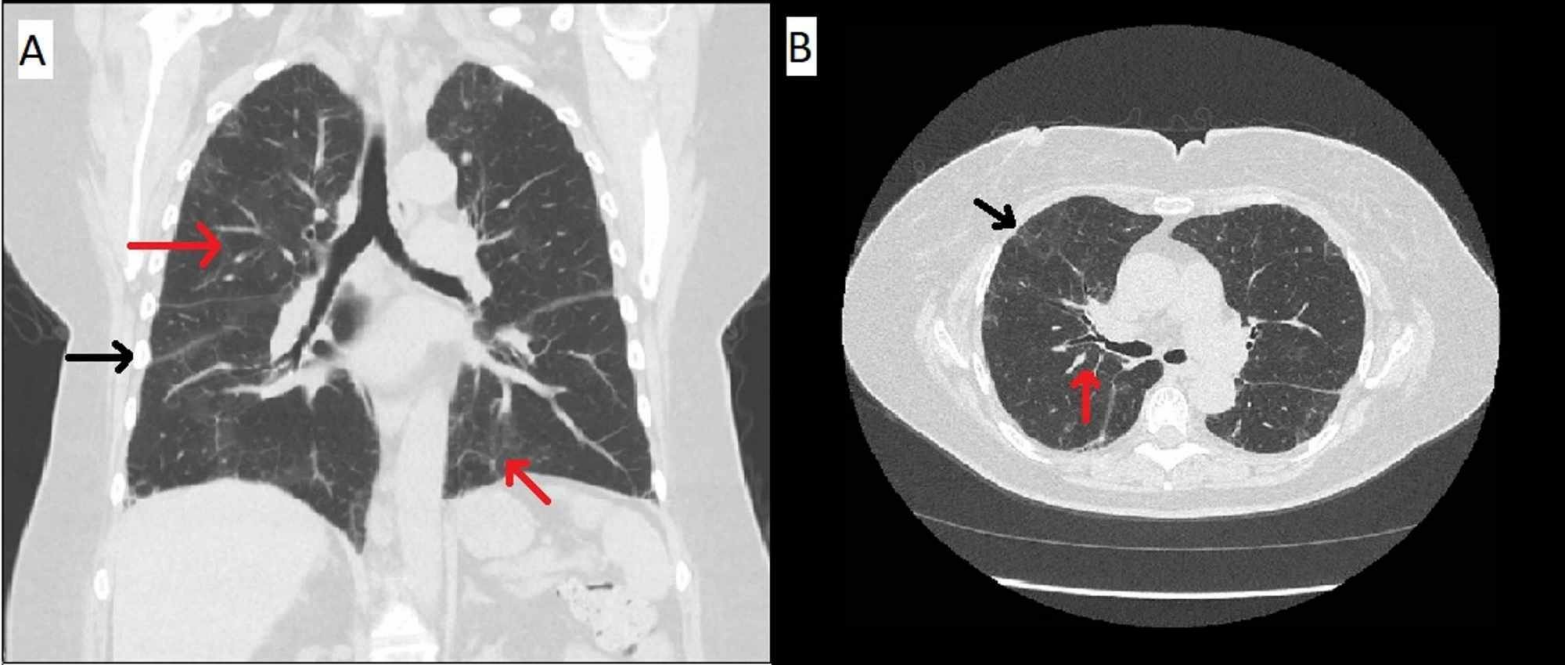
CT imaging three months after presentation Coronal CT chest imaging (A) and axial CT chest imaging (B) showing improvement of the upper lobe predominant peribronchovascular (red arrows) and subpleural nodular areas of consolidation and groundglass opacities (black arrows).

**Figure 3:**
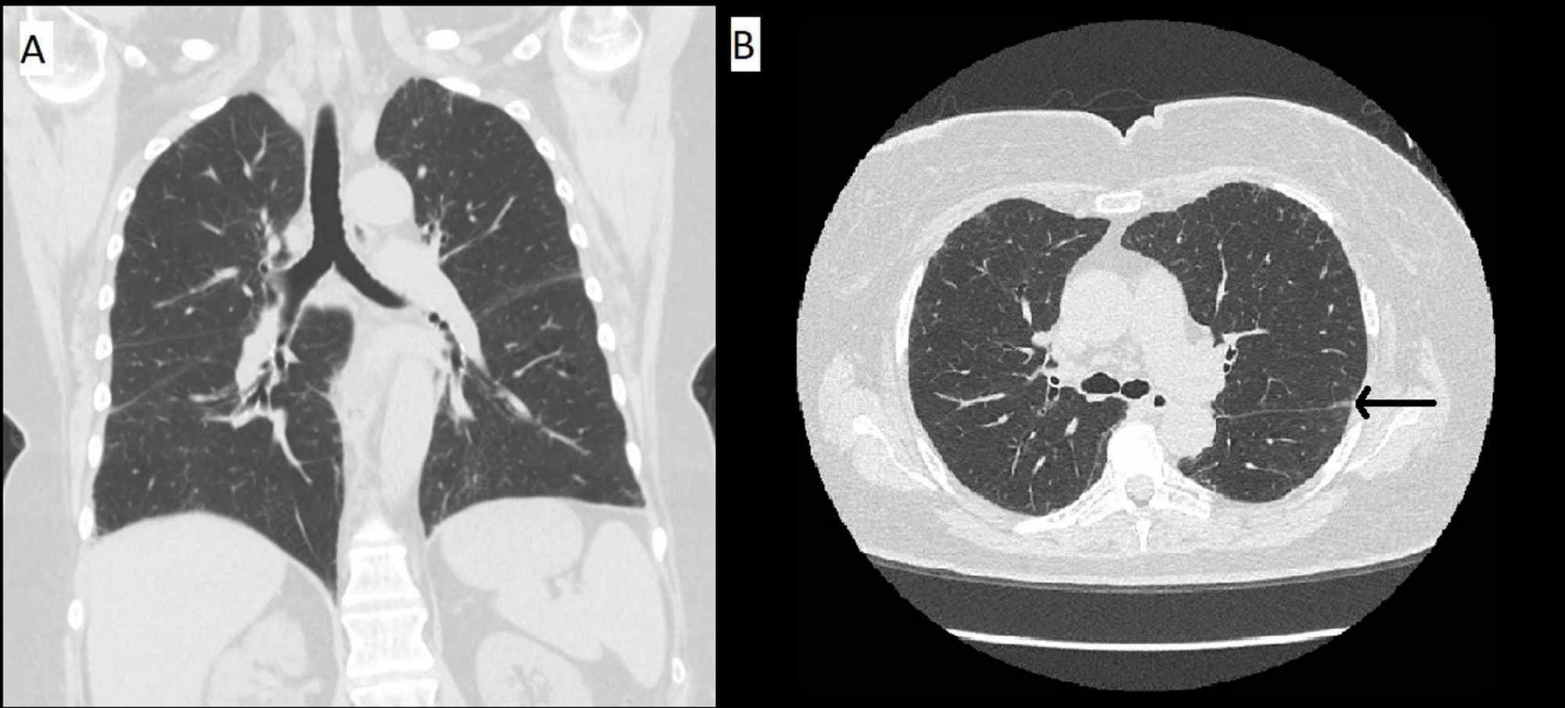
CT imaging nine months after presentation Coronal CT chest imaging (A) and axial CT chest imaging (B) showing continued improvement of parenchymal disease with only subtle residual reticulations and very minimal groundglass (black arrow).

Specifically, follow up HRCT at three months and nine months demonstrated improvement in ground-glass opacities and a decrease in the size of perifissural nodules (Figures 2-3). She was instructed to avoid nitrofurantoin indefinitely to prevent further parenchymal damage.

## Discussion

Most patients who present with pulmonary reactions to nitrofurantoin are women [6-8]. This inherently makes sense, as women are more likely to be diagnosed with urinary tract infections and be repeatedly exposed to the drug. Pulmonary adverse reactions are further classified based on acute versus chronic exposure. Acute adverse reactions are more common when compared to chronic [9]. Additionally women in their sixth and seventh decades of life more prone to developing each, respectively [7].

Acute hypersensitivity pneumonitis presents on average 8.7 days after initiation of therapy according to a small case series [8]. Fever, dyspnea, and cough are common presenting symptoms. Objective findings include crackles on pulmonary auscultation, peripheral eosinophilia, leukocytosis, elevated erythrocyte sedimentation rate (ESR), and consolidation with pleural effusion(s) on chest X-ray [8,10]. Diagnosis is based on history, exclusion of infectious and other common cardiopulmonary diseases (heart failure, asthma, chronic obstructive pulmonary disease), and temporal relationship to nitrofurantoin. Treatment involves immediate cessation of nitrofurantoin, supportive care, appropriate documentation as an allergy, and instruction to avoid nitrofurantoin indefinitely as repeated exposure can elicit a faster and more robust reaction [8,11]. Additionally, while some literature suggest the use of glucocorticoids as an adjunct, their benefit has not been proven [6].

Chronic pneumonitis is most commonly observed in women in their seventh decade who are on long-term therapy [6]. Symptoms associated include dyspnea, dry cough, and fatigue. As previously discussed, fever is not commonly associated with chronic pneumonitis and its presence should prompt providers to search for alternate etiologies on presentation. The insidious onset of symptoms often leads to a lag in diagnosis, as demonstrated in this case, with the majority of patients being on long-term antibiotic therapy for over a year prior to the cessation of the offending agent [6,12].

Diagnosis of chronic pneumonitis is based on history, consideration of other more common etiologies (hypersensitivity pneumonitis, sarcoidosis, rheumatologic disease, and other causes of interstitial lung disease), and clinical response after discontinuation of the offending agent. Physical exam findings are non-specific and can include inspiratory crackles, clubbing, +/- hypoxemia [6]. Imaging findings in chronic pneumonitis include most commonly a non-dominant pattern of bilateral ground-glass opacities versus less common bilateral asymmetric areas of consolidation with patchy ground-glass opacities versus rare honeycombing [6,10,13,14]. Of note, pleural effusions are uncommon in chronic pneumonitis as compared to acute hypersensitivity.

Chronic nitrofurantoin use is associated with a higher risk of parenchymal injury. In one retrospective, matched-cohort study of 13,421 participants. chronic nitrofurantoin therapy had an adjusted risk ratio of 1.53 (CI = 1.04-2.24) [15]. Unfortunately biopsy is not often performed prior to diagnosis and there is no pathognomonic finding for nitrofurantoin-induced lung injury [12,16]. Fortunately, severe toxicities are rather rare, occurring in one of 511 patients (0.2%; 95% CI <0.01% to 1.2%) when reviewing seventeen randomized controlled trials [9]. Clinical improvement typically is seen in weeks to months; however, a majority of patients will have some residual lung disease [6,14].

## Conclusions

Nitrofurantoin is a bactericidal antibiotic that acts on multiple biochemical pathways of the bacterial cell. It is used as first-line, empiric therapy for uncomplicated cystitis as well as daily for prevention for recurrent urinary tract infections. Because of this, the majority of patients that will have an adverse event related to nitrofurantoin are women. Acute hypersensitivity typically presents around day eight or nine of exposure to the drug. It presents with fever, dyspnea, and cough. Labs and imaging display a peripheral eosinophilia, leukocytosis, elevated ESR, and consolidation with pleural effusion on chest X-ray. The backbone of treatment is the cessation of nitrofurantoin therapy. While acute reactions are more common, it is important to remain vigilant for chronic manifestations. When evaluating interstitial lung disease, a thorough medical history is paramount, as many pulmonary toxicities due to medications are reversible if identified early. Our patient’s history of progressively worsening dyspnea on exertion with concurrent, daily nitrofurantoin use fit with a diagnosis of chronic pneumonitis. Our patient demonstrated a peribronchovascular distribution likely consistent with organizing pneumonia, which has rarely been described in the literature. After undergoing extensive work up to rule out infectious, rheumatologic, and other cardiopulmonary disorders, she was diagnosed with nitrofurantoin-induced lung injury. Her nitrofurantoin was stopped and she had subjective improvement, as well as near-resolution of HRCT abnormalities.
